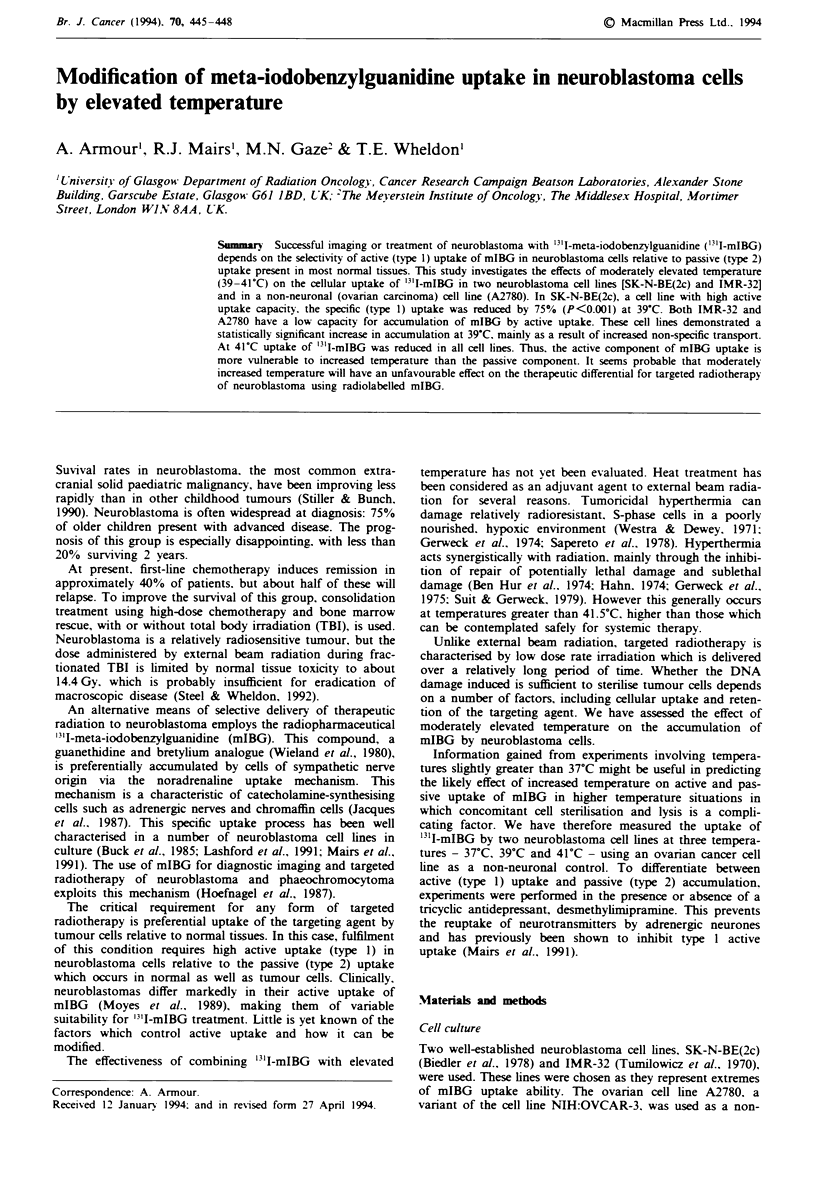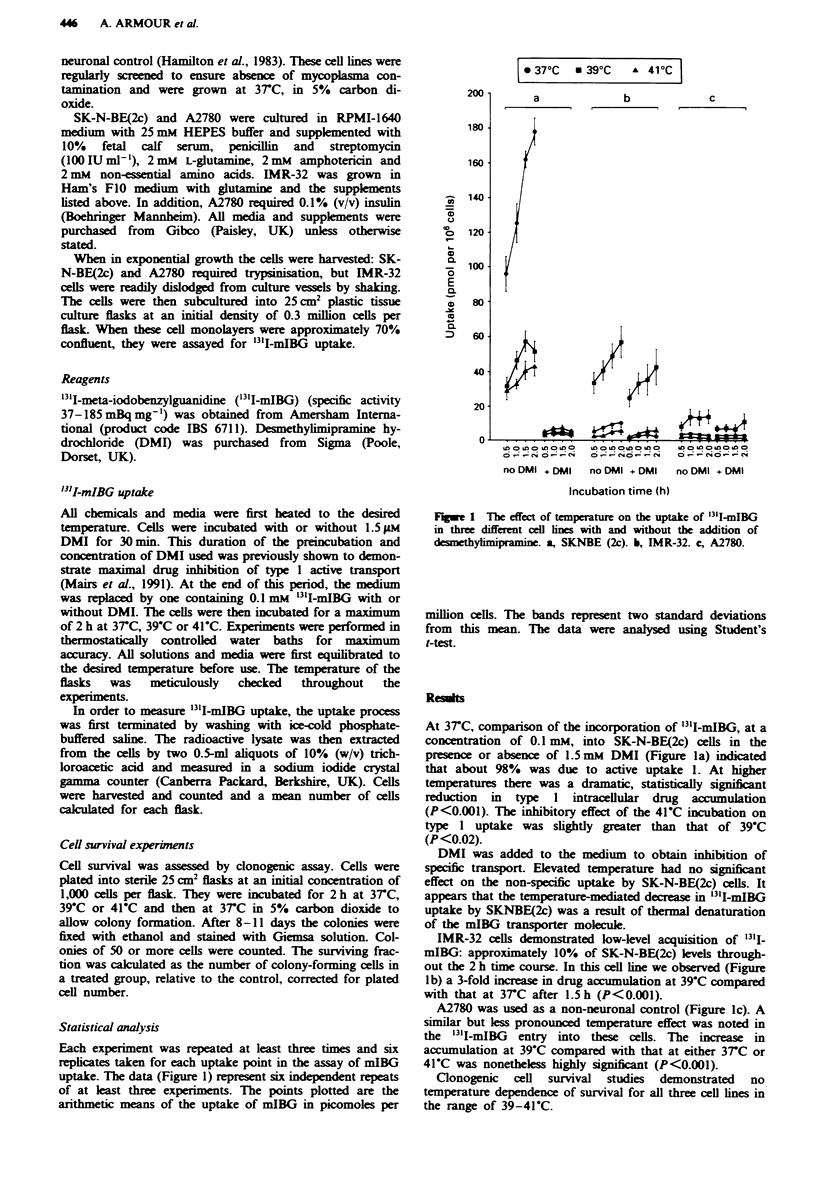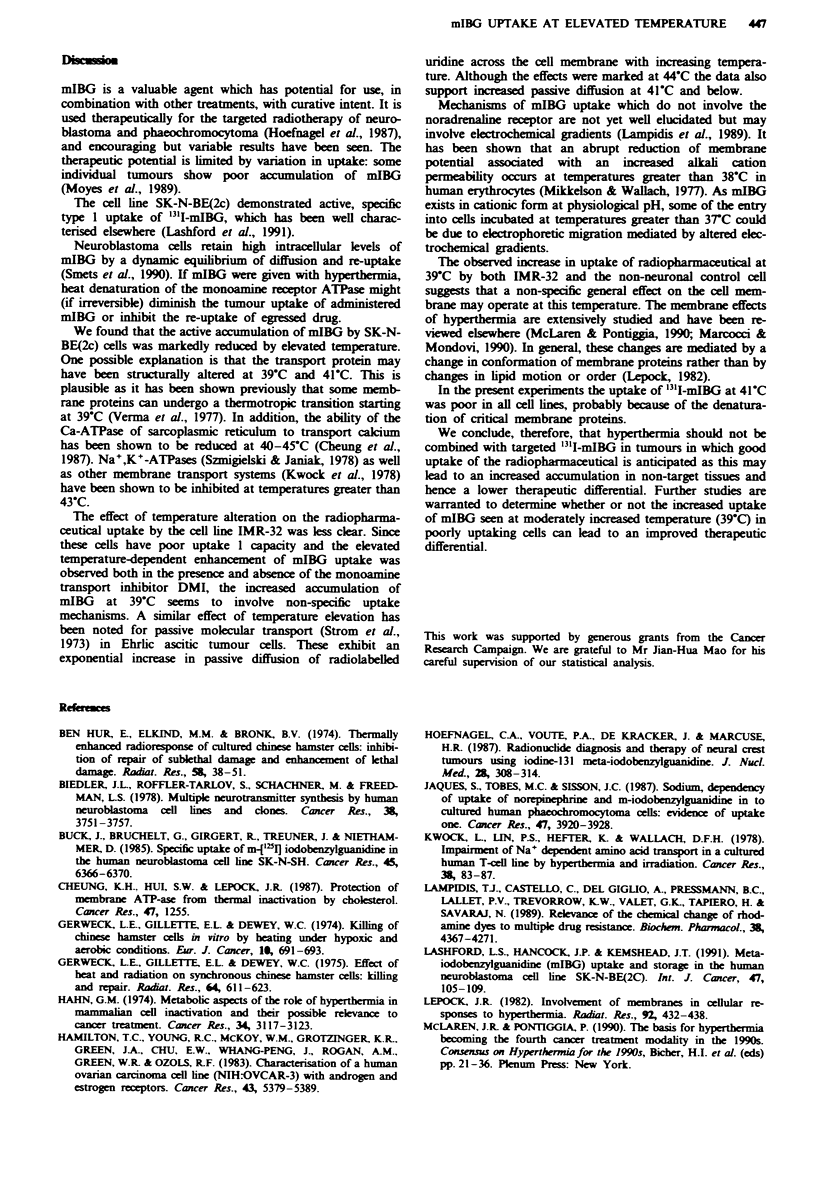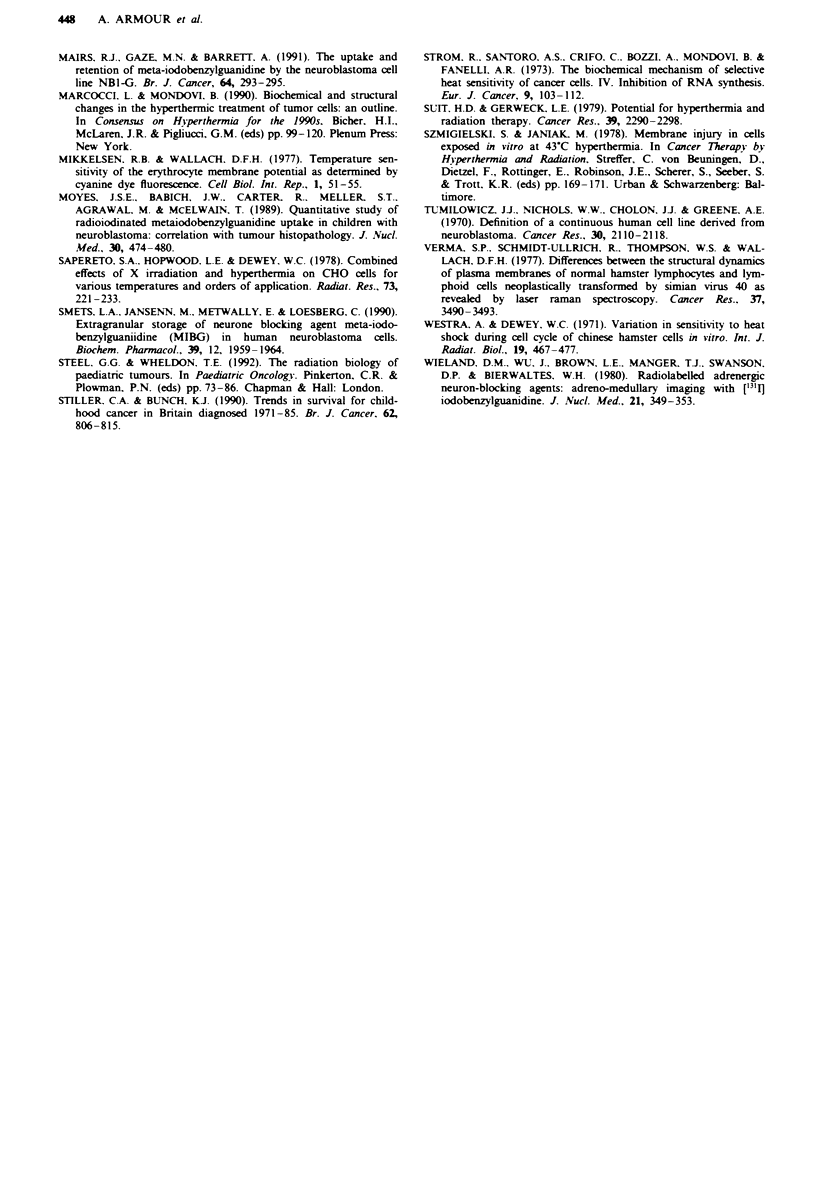# Modification of meta-iodobenzylguanidine uptake in neuroblastoma cells by elevated temperature.

**DOI:** 10.1038/bjc.1994.325

**Published:** 1994-09

**Authors:** A. Armour, R. J. Mairs, M. N. Gaze, T. E. Wheldon

**Affiliations:** University of Glasgow, Department of Radiation Oncology, Cancer Research Campaign Beatson Laboratories, UK.

## Abstract

Successful imaging or treatment of neuroblastoma with 131I-meta-iodobenzylguanidine (131I-mIBG) depends on the selectivity of active (type 1) uptake of mIBG in neuroblastoma cells relative to passive (type 2) uptake present in most normal tissues. This study investigates the effects of moderately elevated temperature (39-41 degrees C) on the cellular uptake of 131I-mIBG in two neuroblastoma cell lines [SK-N-BE(2c) and IMR-32] and in a non-neuronal (ovarian carcinoma) cell line (A2780). In SK-N-BE(2c), a cell line with high active uptake capacity, the specific (type 1) uptake was reduced by 75% (P < 0.001) at 39 degrees C. Both IMR-32 and A2780 have a low capacity for accumulation of mIBG by active uptake. These cell lines demonstrated a statistically significant increase in accumulation at 39 degrees C, mainly as a result of increased non-specific transport. At 41 degrees C uptake of 131I-mIBG was reduced in all cell lines. Thus, the active component of mIBG uptake is more vulnerable to increased temperature than the passive component. It seems probable that moderately increased temperature will have an unfavourable effect on the therapeutic differential for targeted radiotherapy of neuroblastoma using radiolabelled mIBG.


Br. J. Cancer (1994), 70, 445-448 © Macmillan Press Ltd., 1994

### Modification of meta-iodobenzylguanidine uptake in neuroblastoma cells by elevated temperature

A. Armour[1], R.J. Mairs[1], M.N. Gaze[2] & T.E. Wheldon[1]

[1]University of Glasgow Department of Radiation Oncology, Cancer Research Campaign Beatson Laboratories, Alexander Stone Building, Garscube Estate, Glasgow G61 1BD, UK; [2]The Meyerstein Institute of Oncology, The Middlesex Hospital, Mortimer Street, London W1N 8AA, UK.

**Summary** Successful imaging or treatment of neuroblastoma with $^{131}$I-meta-iodobenzylguanidine ($^{131}$I-mIBG) depends on the selectivity of active (type 1) uptake of mIBG in neuroblastoma cells relative to passive (type 2) uptake present in most normal tissues. This study investigates the effects of moderately elevated temperature (39-41°C) on the cellular uptake of $^{131}$I-mIBG in two neuroblastoma cell lines [SK-N-BE(2c) and IMR-32] and in a non-neuronal (ovarian carcinoma) cell line (A2780). In SK-N-BE(2c), a cell line with high active uptake capacity, the specific (type 1) uptake was reduced by 75% ($P<0.001$) at 39°C. Both IMR-32 and A2780 have a low capacity for accumulation of mIBG by active uptake. These cell lines demonstrated a statistically significant increase in accumulation at 39°C, mainly as a result of increased non-specific transport. At 41°C uptake of $^{131}$I-mIBG was reduced in all cell lines. Thus, the active component of mIBG uptake is more vulnerable to increased temperature than the passive component. It seems probable that moderately increased temperature will have an unfavourable effect on the therapeutic differential for targeted radiotherapy of neuroblastoma using radiolabelled mIBG.

Suvival rates in neuroblastoma, the most common extracranial solid paediatric malignancy, have been improving less rapidly than in other childhood tumours (Stiller & Bunch, 1990). Neuroblastoma is often widespread at diagnosis: 75% of older children present with advanced disease. The prognosis of this group is especially disappointing, with less than 20% surviving 2 years.

At present, first-line chemotherapy induces remission in approximately 40% of patients, but about half of these will relapse. To improve the survival of this group, consolidation treatment using high-dose chemotherapy and bone marrow rescue, with or without total body irradiation (TBI), is used. Neuroblastoma is a relatively radiosensitive tumour, but the dose administered by external beam radiation during fractionated TBI is limited by normal tissue toxicity to about 14.4 Gy, which is probably insufficient for eradication of macroscopic disease (Steel & Wheldon, 1992).

An alternative means of selective delivery of therapeutic radiation to neuroblastoma employs the radiopharmaceutical $^{131}$I-meta-iodobenzylguanidine (mIBG). This compound, a guanethidine and bretylium analogue (Wieland *et al.*, 1980), is preferentially accumulated by cells of sympathetic nerve origin via the noradrenaline uptake mechanism. This mechanism is a characteristic of catecholamine-synthesising cells such as adrenergic nerves and chromaffin cells (Jacques *et al.*, 1987). This specific uptake process has been well characterised in a number of neuroblastoma cell lines in culture (Buck *et al.*, 1985; Lashford *et al.*, 1991; Mairs *et al.*, 1991). The use of mIBG for diagnostic imaging and targeted radiotherapy of neuroblastoma and phaeochromocytoma exploits this mechanism (Hoefnagel *et al.*, 1987).

The critical requirement for any form of targeted radiotherapy is preferential uptake of the targeting agent by tumour cells relative to normal tissues. In this case, fulfilment of this condition requires high active uptake (type 1) in neuroblastoma cells relative to the passive (type 2) uptake which occurs in normal as well as tumour cells. Clinically, neuroblastomas differ markedly in their active uptake of mIBG (Moyes *et al.*, 1989), making them of variable suitability for $^{131}$I-mIBG treatment. Little is yet known of the factors which control active uptake and how it can be modified.

The effectiveness of combining $^{131}$I-mIBG with elevated temperature has not yet been evaluated. Heat treatment has been considered as an adjuvant agent to external beam radiation for several reasons. Tumoricidal hyperthermia can damage relatively radioresistant, S-phase cells in a poorly nourished, hypoxic environment (Westra & Dewey, 1971; Gerweck *et al.*, 1974; Sapereto *et al.*, 1978). Hyperthermia acts synergistically with radiation, mainly through the inhibition of repair of potentially lethal damage and sublethal damage (Ben Hur *et al.*, 1974; Hahn, 1974; Gerweck *et al.*, 1975; Suit & Gerweck, 1979). However this generally occurs at temperatures greater than 41.5°C, higher than those which can be contemplated safely for systemic therapy.

Unlike external beam radiation, targeted radiotherapy is characterised by low dose rate irradiation which is delivered over a relatively long period of time. Whether the DNA damage induced is sufficient to sterilise tumour cells depends on a number of factors, including cellular uptake and retention of the targeting agent. We have assessed the effect of moderately elevated temperature on the accumulation of mIBG by neuroblastoma cells.

Information gained from experiments involving temperatures slightly greater than 37°C might be useful in predicting the likely effect of increased temperature on active and passive uptake of mIBG in higher temperature situations in which concomitant cell sterilisation and lysis is a complicating factor. We have therefore measured the uptake of $^{131}$I-mIBG by two neuroblastoma cell lines at three temperatures - 37°C, 39°C and 41°C - using an ovarian cancer cell line as a non-neuronal control. To differentiate between active (type 1) uptake and passive (type 2) accumulation, experiments were performed in the presence or absence of a tricyclic antidepressant, desmethylimipramine. This prevents the reuptake of neurotransmitters by adrenergic neurones and has previously been shown to inhibit type 1 active uptake (Mairs *et al.*, 1991).

### Materials and methods

### Cell culture

Two well-established neuroblastoma cell lines, SK-N-BE(2c) (Biedler *et al.*, 1978) and IMR-32 (Tumilowicz *et al.*, 1970), were used. These lines were chosen as they represent extremes of mIBG uptake ability. The ovarian cell line A2780, a variant of the cell line NIH:OVCAR-3, was used as a non-

Correspondence: A. Armour.

Received 12 January 1994; and in revised form 27 April 1994.

neuronal control (Hamilton et al., 1983). These cell lines were regularly screened to ensure absence of mycoplasma contamination and were grown at 37°C, in 5% carbon dioxide.

SK-N-BE(2c) and A2780 were cultured in RPMI-1640 medium with 25 mM HEPES buffer and supplemented with 10% fetal calf serum, penicillin and streptomycin (100 IU $ml^{-1}$), 2 mM L-glutamine, 2 mM amphotericin and 2 mM non-essential amino acids. IMR-32 was grown in Ham's F10 medium with glutamine and the supplements listed above. In addition, A2780 required 0.1% (v/v) insulin (Boehringer Mannheim). All media and supplements were purchased from Gibco (Paisley, UK) unless otherwise stated.

When in exponential growth the cells were harvested: SK-N-BE(2c) and A2780 required trypsinisation, but IMR-32 cells were readily dislodged from culture vessels by shaking. The cells were then subcultured into 25 $cm^2$ plastic tissue culture flasks at an initial density of 0.3 million cells per flask. When these cell monolayers were approximately 70% confluent, they were assayed for $^{131}$I-mIBG uptake.

### *Reagents*

$^{131}$I-meta-iodobenzylguanidine ($^{131}$I-mIBG) (specific activity 37–185 mBq $mg^{-1}$) was obtained from Amersham International (product code IBS 6711). Desmethylimipramine hydrochloride (DMI) was purchased from Sigma (Poole, Dorset, UK).

### *$^{131}$I-mIBG uptake*

All chemicals and media were first heated to the desired temperature. Cells were incubated with or without 1.5 μM DMI for 30 min. This duration of the preincubation and concentration of DMI used was previously shown to demonstrate maximal drug inhibition of type 1 active transport (Mairs et al., 1991). At the end of this period, the medium was replaced by one containing 0.1 mM $^{131}$I-mIBG with or without DMI. The cells were then incubated for a maximum of 2 h at 37°C, 39°C or 41°C. Experiments were performed in thermostatically controlled water baths for maximum accuracy. All solutions and media were first equilibrated to the desired temperature before use. The temperature of the flasks was meticulously checked throughout the experiments.

In order to measure $^{131}$I-mIBG uptake, the uptake process was first terminated by washing with ice-cold phosphate-buffered saline. The radioactive lysate was then extracted from the cells by two 0.5-ml aliquots of 10% (w/v) trichloroacetic acid and measured in a sodium iodide crystal gamma counter (Canberra Packard, Berkshire, UK). Cells were harvested and counted and a mean number of cells calculated for each flask.

### *Cell survival experiments*

Cell survival was assessed by clonogenic assay. Cells were plated into sterile 25 $cm^2$ flasks at an initial concentration of 1,000 cells per flask. They were incubated for 2 h at 37°C, 39°C or 41°C and then at 37°C in 5% carbon dioxide to allow colony formation. After 8–11 days the colonies were fixed with ethanol and stained with Giemsa solution. Colonies of 50 or more cells were counted. The surviving fraction was calculated as the number of colony-forming cells in a treated group, relative to the control, corrected for plated cell number.

### *Statistical analysis*

Each experiment was repeated at least three times and six replicates taken for each uptake point in the assay of mIBG uptake. The data (Figure 1) represent six independent repeats of at least three experiments. The points plotted are the arithmetic means of the uptake of mIBG in picomoles per million cells. The bands represent two standard deviations from this mean. The data were analysed using Student's *t*-test.

**Figure 1** The effect of temperature on the uptake of $^{131}$I-mIBG in three different cell lines with and without the addition of desmethylimipramine. **a**, SKNBE (2c). **b**, IMR-32. **c**, A2780.

### Results

At 37°C, comparison of the incorporation of $^{131}$I-mIBG, at a concentration of 0.1 mM, into SK-N-BE(2c) cells in the presence or absence of 1.5 mM DMI (Figure 1a) indicated that about 98% was due to active uptake 1. At higher temperatures there was a dramatic, statistically significant reduction in type 1 intracellular drug accumulation ($P<0.001$). The inhibitory effect of the 41°C incubation on type 1 uptake was slightly greater than that of 39°C ($P<0.02$).

DMI was added to the medium to obtain inhibition of specific transport. Elevated temperature had no significant effect on the non-specific uptake by SK-N-BE(2c) cells. It appears that the temperature-mediated decrease in $^{131}$I-mIBG uptake by SKNBE(2c) was a result of thermal denaturation of the mIBG transporter molecule.

IMR-32 cells demonstrated low-level acquisition of $^{131}$I-mIBG: approximately 10% of SK-N-BE(2c) levels throughout the 2 h time course. In this cell line we observed (Figure 1b) a 3-fold increase in drug accumulation at 39°C compared with that at 37°C after 1.5 h ($P<0.001$).

A2780 was used as a non-neuronal control (Figure 1c). A similar but less pronounced temperature effect was noted in the $^{131}$I-mIBG entry into these cells. The increase in accumulation at 39°C compared with that at either 37°C or 41°C was nonetheless highly significant ($P<0.001$).

Clonogenic cell survival studies demonstrated no temperature dependence of survival for all three cell lines in the range of 39–41°C.

### Discussion mIBG is a valuable agent which has potential for use, in combination with other treatments, with curative intent. It is used therapeutically for the targeted radiotherapy of neuroblastoma and phaeochromocytoma (Hoefnagel *et al.*, 1987), and encouraging but variable results have been seen. The therapeutic potential is limited by variation in uptake: some individual tumours show poor accumulation of mIBG (Moyes *et al.*, 1989).

The cell line SK-N-BE(2c) demonstrated active, specific type 1 uptake of $^{131}$I-mIBG, which has been well characterised elsewhere (Lashford *et al.*, 1991).

Neuroblastoma cells retain high intracellular levels of mIBG by a dynamic equilibrium of diffusion and re-uptake (Smets *et al.*, 1990). If mIBG were given with hyperthermia, heat denaturation of the monoamine receptor ATPase might (if irreversible) diminish the tumour uptake of administered mIBG or inhibit the re-uptake of egressed drug.

We found that the active accumulation of mIBG by SK-N-BE(2c) cells was markedly reduced by elevated temperature. One possible explanation is that the transport protein may have been structurally altered at 39°C and 41°C. This is plausible as it has been shown previously that some membrane proteins can undergo a thermotropic transition starting at 39°C (Verma *et al.*, 1977). In addition, the ability of the Ca-ATPase of sarcoplasmic reticulum to transport calcium has been shown to be reduced at 40–45°C (Cheung *et al.*, 1987). $Na^+$,$K^+$-ATPases (Szmigielski & Janiak, 1978) as well as other membrane transport systems (Kwock *et al.*, 1978) have been shown to be inhibited at temperatures greater than 43°C.

The effect of temperature alteration on the radiopharmaceutical uptake by the cell line IMR-32 was less clear. Since these cells have poor uptake 1 capacity and the elevated temperature-dependent enhancement of mIBG uptake was observed both in the presence and absence of the monoamine transport inhibitor DMI, the increased accumulation of mIBG at 39°C seems to involve non-specific uptake mechanisms. A similar effect of temperature elevation has been noted for passive molecular transport (Strom *et al.*, 1973) in Ehrlic ascitic tumour cells. These exhibit an exponential increase in passive diffusion of radiolabelled uridine across the cell membrane with increasing temperature. Although the effects were marked at 44°C the data also support increased passive diffusion at 41°C and below.

Mechanisms of mIBG uptake which do not involve the noradrenaline receptor are not yet well elucidated but may involve electrochemical gradients (Lampidis *et al.*, 1989). It has been shown that an abrupt reduction of membrane potential associated with an increased alkali cation permeability occurs at temperatures greater than 38°C in human erythrocytes (Mikkelson & Wallach, 1977). As mIBG exists in cationic form at physiological pH, some of the entry into cells incubated at temperatures greater than 37°C could be due to electrophoretic migration mediated by altered electrochemical gradients.

The observed increase in uptake of radiopharmaceutical at 39°C by both IMR-32 and the non-neuronal control cell suggests that a non-specific general effect on the cell membrane may operate at this temperature. The membrane effects of hyperthermia are extensively studied and have been reviewed elsewhere (McLaren & Pontiggia, 1990; Marcocci & Mondovi, 1990). In general, these changes are mediated by a change in conformation of membrane proteins rather than by changes in lipid motion or order (Lepock, 1982).

In the present experiments the uptake of $^{131}$I-mIBG at 41°C was poor in all cell lines, probably because of the denaturation of critical membrane proteins.

We conclude, therefore, that hyperthermia should not be combined with targeted $^{131}$I-mIBG in tumours in which good uptake of the radiopharmaceutical is anticipated as this may lead to an increased accumulation in non-target tissues and hence a lower therapeutic differential. Further studies are warranted to determine whether or not the increased uptake of mIBG seen at moderately increased temperature (39°C) in poorly uptaking cells can lead to an improved therapeutic differential.

This work was supported by generous grants from the Cancer Research Campaign. We are grateful to Mr Jian-Hua Mao for his careful supervision of our statistical analysis.

### References

BEN HUR, E., ELKIND, M.M. & BRONK, B.V. (1974). Thermally enhanced radioresponse of cultured chinese hamster cells: inhibition of repair of sublethal damage and enhancement of lethal damage. *Radiat. Res.*, **58**, 38–51.

BIEDLER, J.L., ROFFLER-TARLOV, S., SCHACHNER, M. & FREEDMAN, L.S. (1978). Multiple neurotransmitter synthesis by human neuroblastoma cell lines and clones. *Cancer Res.*, **38**, 3751–3757.

BUCK, J., BRUCHELT, G., GIRGERT, R., TREUNER, J. & NIETHAMMER, D. (1985). Specific uptake of m-[$^{125}$I] iodobenzylguanidine in the human neuroblastoma cell line SK-N-SH. *Cancer Res.*, **45**, 6366–6370.

CHEUNG, K.H., HUI, S.W. & LEPOCK, J.R. (1987). Protection of membrane ATP-ase from thermal inactivation by cholesterol. *Cancer Res.*, **47**, 1255.

GERWECK, L.E., GILLETTE, E.L. & DEWEY, W.C. (1974). Killing of chinese hamster cells *in vitro* by heating under hypoxic and aerobic conditions. *Eur. J. Cancer*, **10**, 691–693.

GERWECK, L.E., GILLETTE, E.L. & DEWEY, W.C. (1975). Effect of heat and radiation on synchronous chinese hamster cells: killing and repair. *Radiat. Res.*, **64**, 611–623.

HAHN, G.M. (1974). Metabolic aspects of the role of hyperthermia in mammalian cell inactivation and their possible relevance to cancer treatment. *Cancer Res.*, **34**, 3117–3123.

HAMILTON, T.C., YOUNG, R.C., MCKOY, W.M., GROTZINGER, K.R., GREEN, J.A., CHU, E.W., WHANG-PENG, J., ROGAN, A.M., GREEN, W.R. & OZOLS, R.F. (1983). Characterisation of a human ovarian carcinoma cell line (NIH:OVCAR-3) with androgen and estrogen receptors. *Cancer Res.*, **43**, 5379–5389.

HOEFNAGEL, C.A., VOUTE, P.A., DE KRACKER, J. & MARCUSE, H.R. (1987). Radionuclide diagnosis and therapy of neural crest tumours using iodine-131 meta-iodobenzylguanidine. *J. Nucl. Med.*, **28**, 308–314.

JAQUES, S., TOBES, M.C. & SISSON, J.C. (1987). Sodium, dependency of uptake of norepinephrine and m-iodobenzylguanidine in to cultured human phaeochromocytoma cells: evidence of uptake one. *Cancer Res.*, **47**, 3920–3928.

KWOCK, L., LIN, P.S., HEFTER, K. & WALLACH, D.F.H. (1978). Impairment of $Na^+$ dependent amino acid transport in a cultured human T-cell line by hyperthermia and irradiation. *Cancer Res.*, **38**, 83–87.

LAMPIDIS, T.J., CASTELLO, C., DEL GIGLIO, A., PRESSMANN, B.C., LALLET, P.V., TREVORROW, K.W., VALET, G.K., TAPIERO, H. & SAVARAJ, N. (1989). Relevance of the chemical change of rhodamine dyes to multiple drug resistance. *Biochem. Pharmacol.*, **38**, 4367–4271.

LASHFORD, L.S., HANCOCK, J.P. & KEMSHEAD, J.T. (1991). Meta-iodobenzylguanidine (mIBG) uptake and storage in the human neuroblastoma cell line SK-N-BE(2C). *Int. J. Cancer*, **47**, 105–109.

LEPOCK, J.R. (1982). Involvement of membranes in cellular responses to hyperthermia. *Radiat. Res.*, **92**, 432–438.

MCLAREN, J.R. & PONTIGGIA, P. (1990). The basis for hyperthermia becoming the fourth cancer treatment modality in the 1990s. *Consensus on Hyperthermia for the 1990s*, Bicher, H.I. *et al.* (eds) pp. 21–36. Plenum Press: New York.

MAIRS, R.J., GAZE, M.N. & BARRETT, A. (1991). The uptake and retention of meta-iodobenzylguanidine by the neuroblastoma cell line NB1-G. *Br. J. Cancer*, **64**, 293-295.

MARCOCCI, L. & MONDOVI, B. (1990). Biochemical and structural changes in the hyperthermic treatment of tumor cells: an outline. In *Consensus on Hyperthermia for the 1990s*, Bicher, H.I., McLaren, J.R. & Pigliucci, G.M. (eds) pp. 99-120. Plenum Press: New York.

MIKKELSEN, R.B. & WALLACH, D.F.H. (1977). Temperature sensitivity of the erythrocyte membrane potential as determined by cyanine dye fluorescence. *Cell Biol. Int. Rep.*, **1**, 51-55.

MOYES, J.S.E., BABICH, J.W., CARTER, R., MELLER, S.T., AGRAWAL, M. & McELWAIN, T. (1989). Quantitative study of radioiodinated metaiodobenzylguanidine uptake in children with neuroblastoma: correlation with tumour histopathology. *J. Nucl. Med.*, **30**, 474-480.

SAPERETO, S.A., HOPWOOD, L.E. & DEWEY, W.C. (1978). Combined effects of X irradiation and hyperthermia on CHO cells for various temperatures and orders of application. *Radiat. Res.*, **73**, 221-233.

SMETS, L.A., JANSENN, M., METWALLY, E. & LOESBERG, C. (1990). Extragranular storage of neurone blocking agent meta-iodobenzylguaniidine (MIBG) in human neuroblastoma cells. *Biochem. Pharmacol.*, **39**, 12, 1959-1964.

STEEL, G.G. & WHELDON, T.E. (1992). The radiation biology of paediatric tumours. In *Paediatric Oncology*. Pinkerton, C.R. & Plowman, P.N. (eds) pp. 73-86. Chapman & Hall: London.

STILLER, C.A. & BUNCH, K.J. (1990). Trends in survival for childhood cancer in Britain diagnosed 1971-85. *Br. J. Cancer*, **62**, 806-815.

STROM, R., SANTORO, A.S., CRIFO, C., BOZZI, A., MONDOVI, B. & FANELLI, A.R. (1973). The biochemical mechanism of selective heat sensitivity of cancer cells. IV. Inhibition of RNA synthesis. *Eur. J. Cancer*, **9**, 103-112.

SUIT, H.D. & GERWECK, L.E. (1979). Potential for hyperthermia and radiation therapy. *Cancer Res.*, **39**, 2290-2298.

SZMIGIELSKI, S. & JANIAK, M. (1978). Membrane injury in cells exposed *in vitro* at 43°C hyperthermia. In *Cancer Therapy by Hyperthermia and Radiation*, Streffer, C. von Beuningen, D., Dietzel, F., Rottinger, E., Robinson, J.E., Scherer, S., Seeber, S. & Trott, K.R. (eds) pp. 169-171. Urban & Schwarzenberg: Baltimore.

TUMILOWICZ, J.J., NICHOLS, W.W., CHOLON, J.J. & GREENE, A.E. (1970). Definition of a continuous human cell line derived from neuroblastoma. *Cancer Res.*, **30**, 2110-2118.

VERMA, S.P., SCHMIDT-ULLRICH, R., THOMPSON, W.S. & WALLACH, D.F.H. (1977). Differences between the structural dynamics of plasma membranes of normal hamster lymphocytes and lymphoid cells neoplastically transformed by simian virus 40 as revealed by laser raman spectroscopy. *Cancer Res.*, **37**, 3490-3493.

WESTRA, A. & DEWEY, W.C. (1971). Variation in sensitivity to heat shock during cell cycle of chinese hamster cells *in vitro*. *Int. J. Radiat. Biol.*, **19**, 467-477.

WIELAND, D.M., WU, J., BROWN, L.E., MANGER, T.J., SWANSON, D.P. & BIERWALTES, W.H. (1980). Radiolabelled adrenergic neuron-blocking agents: adreno-medullary imaging with [$^{131}$I] iodobenzylguanidine. *J. Nucl. Med.*, **21**, 349-353.